# Rise of the Earliest Tetrapods: An Early Devonian Origin from Marine Environment

**DOI:** 10.1371/journal.pone.0022136

**Published:** 2011-07-14

**Authors:** David George, Alain Blieck

**Affiliations:** 1 Department of Biotechnology, St. Peter's Engineering College, Chennai, Tamil Nadu, India; 2 Université Lille 1, Sciences de la Terre, FRE 3298 du CNRS Géosystèmes, Villeneuve d'Ascq, France; University of Western Ontario, Canada

## Abstract

Tetrapod fossil tracks are known from the Middle Devonian (Eifelian at ca. 397 million years ago - MYA), and their earliest bony remains from the Upper Devonian (Frasnian at 375–385 MYA). Tetrapods are now generally considered to have colonized land during the Carboniferous (i.e., after 359 MYA), which is considered to be one of the major events in the history of life. Our analysis on tetrapod evolution was performed using molecular data consisting of 13 proteins from 17 species and different paleontological data. The analysis on the molecular data was performed with the program TreeSAAP and the results were analyzed to see if they had implications on the paleontological data collected. The results have shown that tetrapods evolved from marine environments during times of higher oxygen levels. The change in environmental conditions played a major role in their evolution. According to our analysis this evolution occurred at about 397–416 MYA during the Early Devonian unlike previously thought. This idea is supported by various environmental factors such as sea levels and oxygen rate, and biotic factors such as biodiversity of arthropods and coral reefs. The molecular data also strongly supports lungfish as tetrapod's closest living relative.

## Introduction

Terrestrialization may be defined as the series of processes (adaptation) that makes an aquatic organism capable of living and sustaining itself on land. It is usually considered as one of the most important events in the evolutionary history of life on Earth. It has occurred several times, that is among primitive organisms (bacteria, protists, fungi…), plants, invertebrates and vertebrates. Here we focus on vertebrate terrestrialization also known as evolution of fish to tetrapods. Lobe-finned fishes (Sarcopterygii) were a highly successful group during the Devonian, between ca. 416 and 359 million years ago (MYA). According to the most recent discoveries and ideas, terrestrialization of vertebrates has occurred in two steps: 1) the first tetrapods diverged from sarcopterygians during the Frasnian (about 375–385 MYA) or earlier in aquatic environments [1], [2], [3]; 2) this was followed by adaptation to terrestrial life much later in the earliest Carboniferous, about 345–359 MYA. Today only three groups of sarcopterygians survive, namely Tetrapoda, Dipnoi (lungfishes), and Actinistia (coelacanths). Tetrapods include about 21,100 extant species and a much greater number of extinct species; only six species of lungfishes and two species of coelacanths exist to-day, but both groups were much more abundant and diverse in the Devonian. It is generally accepted that elpistostegids, a group of extinct sarcopterygian fishes, are the closest relatives (sister-group) of tetrapods [4]. The early vertebrate expert community very often follows the idea originally proposed by A. S. Romer (e.g. [5]) that early tetrapod life occurred in freshwater under the “Drying Pond” scenario where tetrapods evolved from lobe-finned fishes driven onto the land by drought. A modern version of this scenario is that tetrapods evolved from the elpistostegids, probably in brackish to freshwater environments, in response to the modification of their environment [6]. This scenario has however been strongly contradicted as early as the 1950s (refs in [5]) and replaced by the idea that the transition from fish to tetrapod occurred in marine to land/sea transitional environments (tidal, intertidal or lagoonal zones) [7], [8], [9], [10], [11]. Devonian tetrapods and elpistostegids have been found in a wide range of geographic localities, including the Old Red Sandstone Continent, North China, and East Gondwana. This wide range could be due to marine tolerance. The first tetrapods, like their immediate piscine sister taxa, were capable of marine dispersal, thus explaining the widespread global distribution achieved in the Frasnian [12], [13]. The recent discovery of tetrapod tracks from Poland [11] also suggests that the earliest evolution of tetrapods could have taken place in marine to land/sea transitional environments. Study of such relationships between living organisms and environmental conditions at global scale is generally known as geobiology (biosphere-geosphere interactions).

Attempts were made for a long time to determine whether or not sister relationships in the phylogenetic tree of vertebrates exist between tetrapods and lungfishes, tetrapods and coelacanths, or lungfishes and coelacanths. The mitochondrial [14], [15], ribosomal [16], and nuclear [17], [18] encoded sequences have been collected with the specific goal of resolving the relationships among living sarcopterygians, but the available molecular data has not provided complete resolution to whether lungfishes are the closest living relatives to tetrapods, or coelacanths are the closest, or the third case where both coelacanths and lungfishes could be equally related to tetrapods. The proteins used in this analysis on dipnoans, coelacanths and basal tetrapods (amphibians) are the 13 proteins synthesized by the mitochondrial genome. Modifications in the mitochondrial protein coding genes which are involved in oxidative phosphorylation (a process in cell metabolism by which respiratory enzymes in the mitochondria synthesize ATP, which is used to produce energy) can directly influence metabolic performance of an organism. Because of the importance of this biochemical pathway, evaluating selective pressures acting on mtDNA (mitochondrial DNA) proteins could provide key insight into the adaptive evolution of the mtDNA genome. One important goal of the present paper is to compare molecular to paleontological data in order to improve our view of early steps of tetrapod evolution, and place this process into a geobiological approach.

## Materials and Methods

The 13 proteins synthesized by the mitochondrial genome of 17 species (see Table S1) were obtained from the NCBI (National Centre for Biotechnology Information). Only 17 sequences were used to avoid noise (convergent evolution) in the genes used, since amphibians seem to have a higher rate of nucleotide substitution while coelacanths and Australian lungfish have a lower rate. This will cause many problems when trying to compute any phylogenetic analysis. All the 12 species of tetrapods used were amphibians (in which 6 were salamanders). It was made sure that representatives from all the 3 orders of Amphibia namely Caudata (salamanders, newts, etc.), Anura (frogs, toads), and Gymnophiona (caecilians) were taken in the analysis. Since there is still debate about the relationship between coelacanths, lungfishes and tetrapods, an exact tree cannot be used for the analysis by TreeSAAP (Selection on Amino Acid Properties using phylogenetic Trees) [19]. Hence the analysis was performed by computing the 12 tetrapod species separately, 3 lungfishes and 2 coelacanths. Physicochemical amino acid changes among residues in mitochondrial protein coding genes were identified by the algorithm implemented in TreeSAAP, which compares the observed distribution of physicochemical changes inferred from a phylogenetic tree with an expected distribution based on the assumption of completely random amino acid replacement expected under the condition of selective neutrality. TreeSAAP also helps us to find positive or negative selection in the given sequences (Positive selection indicates that amino acid replacements are being preferred by natural selection, whereas negative selection means they are less frequent than expected by chance and are influenced by negative or purifying selection). This is done by computing the influence of amino acid properties in the given sequences. The positive selection was calculated by taking two different considerations. In the first consideration the values (called z-scores) of the individual amino acid sites were analyzed, and in the second consideration the entire protein sequence values was analyzed. For the calculation of positive selection when the entire protein was taken into analysis, the total sum value of all the individual amino acid sites needs to be calculated, this included the positive selection and the negative selection values of the individual amino acid sites. For example assume a protein has 4 amino acids. Assume the individual amino acid site values are 2, −2, −6, 4. Hence one amino acid site has been influenced by positive selection (any value above 3.09 was considered as positive selection, this value is most commonly used for this program). Now to calculate the positive selection when the whole protein sequence is taken to consideration add all the values (2+(−2)+(−6)+4 = −2). Since −2<3.09 this protein has not undergone positive selection (the detailed z-scores given by TreeSAAP are available on request to the corresponding author). Hence different results are possible when the entire protein sequence and individual amino acid sites are analyzed. Out of the 31 amino acid properties available in the software, only 20 were used in the analysis (see Text S1). This was done to increase the accuracy in detecting protein adaptation and to prevent false indications of protein adaptation. TreeSAAP was implemented by grouping changes into categories from 1 to 8, 1 being the most conservative and 8 being the most radical. When positive selection is detected in lower, more conservative magnitude ranges (categories 1, 2, or 3), the amino acid properties are considered to be under a type of stabilizing selection (here defined as selection that tends to maintain the overall biochemistry of the protein, despite a rate of change that exceeds the rate expected under conditions of chance). Conversely, when positive selection is detected in greater, more radical magnitude ranges (categories 6, 7, or 8), the amino acid property or properties are considered to be under destabilizing selection (here defined as selection that results in radical structural or functional shifts in local regions of the protein). We make the assumption that positive-destabilizing selection represents the unambiguous signature of molecular adaptation because when radical changes are favored by selection, they result in local directional shifts in biochemical function, structure, or both. For such changes to be favored by selection (i.e., for such changes to be more abundant than expected by chance), they must instill an increased level of survival and/or reproductive success in the individuals who possess and propagate them (refer [20] for more details). In this study we choose to focus on amino acid property changes of categories 6, 7, and 8 because they unambiguously indicate a significant change in the protein (See Table S2).

The GEOCARBSULF model [21] was used to know the atmospheric oxygen levels during the Devonian. It is a combination of earlier GEOCARB models for CO_2_ and the isotope mass balance model for O_2_. GEOCARBSULF is a computer model that takes account of the major factors thought to influence atmospheric O_2_ and CO_2_. These models account for “forcings,” which are processes that affect the levels of these gases. Principal forcings for O_2_ are burial of organic matter and pyrite (FeS_2_) in sediments, their weathering on the continents, and rates of metamorphic and volcanic degassing of reduced carbon and sulfur-containing volcanic gases, such as sulfur dioxide.

## Results

### Adaptive Evolution Results

Amino acid properties with signals of strong positive selection accumulated at a rate roughly equivalent to the mutation rate of the gene itself (i.e. mutation rate of ATPase (Adenosine Triphosphate Synthase)>ND (Nicotinamide adenine dinucleotide dehydrogenase)>CYTB (Cytochrome b)>COX (Cytochrome c Oxidase)). This correlation is more pronounced for the protein-coding genes with higher mutation rates, such as ATPase and ND, as is most apparent in analyses of the variation. The best correlation between overall mutation rate and number of sites with radical amino acid changes was observed for NDs, while the existence of several outliers for ATPase slightly reduced the strength of the correlation. The biochemical complexity of the oxidative phosphorylation processes precludes a clear discussion on the functional implications of the amino acid properties that are under selection. The amino acid properties under positive destabilizing selection when the entire protein sequence was taken into consideration for analysis are Solvent accessible reduction ratio, Thermodynamic transfer hydrophobicity. This feature was observed only for the amphibians. No positive destabilizing selection was observed when the entire protein sequence of lungfishes and coelacanths were taken into consideration for analysis. But positive destabilizing selection was observed when individual amino acid sites were taken into consideration. Protein sequences in all the three groups namely amphibians, coelacanths and lungfishes (see Table 1) showed positive selection. Individual amino acid sites influenced by positive destabilizing selection were more similar between lungfishes and amphibians (439 similar sites from 13 proteins) than between coelacanths and amphibians (98 similar sites from 13 proteins). The least similarity was found between lungfishes and coelacanths (16 similar sites from 13 proteins). Please note that the values in Table 1 are NOT to be totaled since the table only lists the major properties, and also a same amino acid site might have been affected by more than one amino acid property, hence if values in Table 1 are added it will give an incorrect higher value (Refer Table S2).

**Table 1:** Total number of similar amino acid sites between the different groups of sarcopterygians which have been affected by positive-destabilizing during cladogenesis.

	A-L	A-C	L-C	A-L-C
pK'	117	26	6	0
R_a_	157	19	7	0
a_n_	2	17	0	17
H_p_	14	9	0	0
H	16	0	1	3
K^o^	62	2	3	2
F	64	13	0	5
H_t_	27	2	0	0
P	15	16	0	3

Abbreviations: A- Amphibians, L- Lungfishes, C- Coelacanths; (pK') - Equilibrium constant (ionization of COOH), (R_a_) - Solvent accessible reduction ratio, (a_n_) - Power to be at the N-terminal, (H_p_) - Surrounding hydrophobicity, (H) - Hydropathy, (K^o^) - Compressibility, (F) - Mean r.m.s. fluctuation displacement, (H_t_) - Thermodynamic transfer hydrophobicity, (P) – Polarity. The table shows clearly that the total number of amino acid sites commonly affected by positive selection is much higher between amphibians and lungfishes (A-L) when compared to the other groupings. Hence we come to a conclusion that amphibians and lungfishes are very closely related. Also pK' and R_a_ have affected the genes to a maximum extent unlike other properties. Please note that only the major amino acid properties are listed in the table (Refer to Table S2 for all the details).

### Geobiological Data

**Figure 1:**
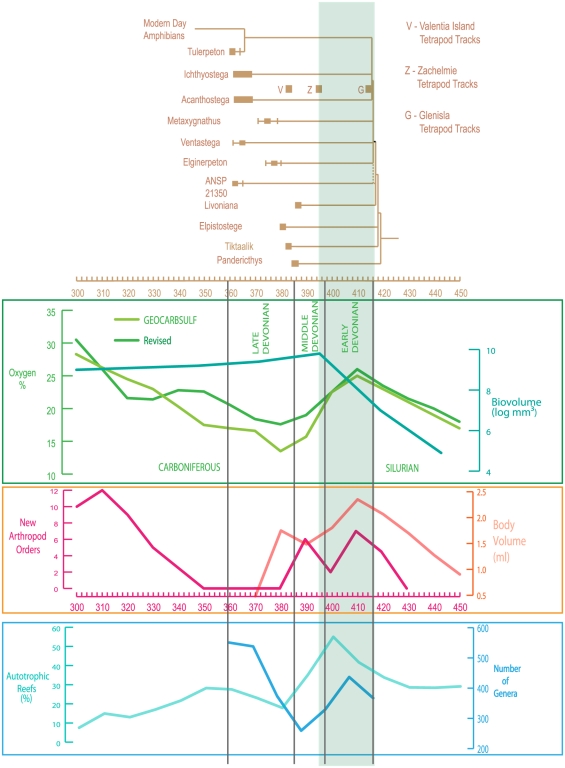
Genus-level biodiversity and phylogenetic relationships of elpistostegid fish and earliest tetrapods, as compared to abiotic and biotic features of Devonian environments. Due to the choosen phylogenetic scheme (after [11]), and the fact that we take into consideration the Glenisla trace fossils from Australia, ghost ranges of basal taxa (elpistostegids, from *Panderichthys* to *Livoniana*) and tetrapods (from ANSP 21350 to the crown group ‘*Tulerpeton* + modern amphibians’) are increased in a significant amount. We use oxygen levels predicted by GEOCARBSULF [21], evolution of arthropod orders [24], evolution of autotrophic reefs [27], body volume of marine invertebrates [26], and genus-level diversity of marine invertebrates [25]. It must be noted that in the highlighted zone of the diagram, the arthropods concerned with are three clades of terrestrial arthropods (myriapods, arachnids, hexapods). Hence the image gives a view of changes in terrestrial and marine species, but giving stress about the changes in marine environment since this is where the tetrapods evolved. The Zachelmie tracks [11] are quite close to the highlighted region and the Glenisla tracks [22] find a satisfactory position amongst the controversy in our image.

Most recent interpretations about the origin of tetrapods and their Devonian representatives lead to conclude that they originated before the Middle Devonian, and probably in the Early Devonian [1], [11], [22]. It is here that the trackway found in the courtyard of Glenisla Homestead, in the Grampians Mountains, western Victoria, Australia [22] takes all its importance [1], [23]. Because of its age (see here below under ‘Discussion’) it does indeed bring a physical argument for an Early Devonian origin of tetrapods. So, higher levels of atmospheric oxygen from the GEOCARBSULF model and the revised model [21] that were detected in the Early Devonian at ca. 397–416 MYA seem to coincide with the “elpistostegid-tetrapod changeover” (*sensu* [11]). Interestingly there seems to be an increase in terrestrial arthropod orders, autotrophic reef biodiversity, marine invertebrate size and genera during the same time [24], [25], [26], [27]. Although coincidence is not necessarily evidence of correlation, it is suggested here that these events were indeed related. Another important finding from the analysis is the re-confirmation of the earliest Carboniferous Romer's Gap as a low oxygen interval [24] although the revised GEOCARBSULF model [21] shows a higher oxygen level than the previous model (Fig. 1). Compared to the Early Devonian genus-level biodiversity of marine invertebrates of about 585 genera, the Early Carboniferous one is below 400 genera [25] which fits with a lower oxygen interval although further analysis would provide better insights and improved clarity to the problem.

An important point to stress is the influence of the fossil record (likelihood of preservation, differences in paleoenvironments, abundance of field prospectionss, etc.) that certainly has an influence on the observed fossil diversity. Additionally it appears that many paleontologists (including the present senior author) had wrong impressions concerning the fossil diversity through time. A single example is given here, viz. the number of genera of reef builders that is higher in the late Early Devonian than in the rest of the Devonian (Fig. 1). It is indeed often said and tought that the most important period of coral reef development has been the Givetian-Frasnian (late Middle to early Late Devonian) time slice when huge reef systems, compared to the present day Australian Great Barrier, were developed in, e.g., Canadian Arctic or Western Australia [28], [29], [30]. However, most recent global evaluations of reef diversity in the Devonian show that this is not the case, the highest mean reef thickness and reef diversity being reached in the late Early Devonian [27], [31]. Such reappraisal for a single group of organisms, if generalized to all terrestrial and aquatic Paleozoic taxa, will certainly give a very different picture from the classical one depicted by, e.g., the ‘Sepkoski Curve’ ([32], and later critical reevaluations such as, e.g., [25], [33]). This very interesting topic is, however, out of the scope of the present paper.

## Discussion

The major amino acid properties affecting the similar regions in the genes of amphibians, lungfishes, and coelacanths (see Table S2) are Equilibrium constant (ionization of COOH), Surrounding hydrophobicity, Power to be at the N-terminal, Solvent accessible reduction ratio, Hydropathy, Compressibility, Mean r.m.s. fluctuation displacement, Thermodynamic transfer hydrophobicity, Polarity. All the above properties were detected as influencing in a positive destabilizing selective direction. Since positive destabilizing selection indicates significant change in the protein, only such changes were taken in account for the analysis. When radical changes are favored by selection, they result in local directional shifts in biochemical function, structure, or both. Increase in Equilibrium constant (ionization of COOH) could influence the efficiency of a protein, interestingly this property would reduce the radical oxygen species production which would increase the longevity of the species since it is generally considered now that increase in radical oxygen species is the main reason for aging [34]. Surrounding hydrophobicity refers to the tendency for the region around the amino acid site in question to interact with water. This is important in our case since the proteins used here are transmembrane proteins. It is similar to hydropathy. The proteins becoming more hydrophobic could mean that many residues get buried inside making the protein more compact. Compressibility is a very important property since it influences the stability of the protein which shows that they have become more stable. Solvent accessible reduction ratio is the property that has mostly affected the proteins. The increase in this value suggests that proteins could have become bulkier and allowing more space for active site formation. The adaptive evolution data shows that Equilibrium constant (ionization of COOH) is the property that has influenced the genes to the second highest extent (Table 1), it drives a more product driven reaction in tetrapod mitochondrial proteins which is why we find a higher value of it affecting the genome.

The levels of oxygen predicted as per GEOCARBSULF [21] has already been studied in relation to tetrapods and arthropods [24]. It is interesting to note that 9 myriapod clades, 4 arachnid clades, and 3 hexapod clades have evolved about 397–416 MYA [24], which seems to confirm the presence of higher levels of oxygen as predicted. Also the increase in distribution of autotrophic reefs [27] could be indicating better formation of reefs in relation to higher levels of oxygen. The diversification of vascular plants [35] and the expansion of high energy predators, including large predatory fish [36], [37], both events of major biological significance, occurred during the same period. An analysis with the use of isotopic composition and concentration of molybdenum in sedimentary rocks [38], and a review of maximum size of organisms through geological time [39] also indicate an increase in oxygen levels in relation to a strong increase in chordate maximum length around 400 MYA. Dahl et al. 's [38] study says that this event could have been the greatest oxygenation (rise in atmospheric oxygen level) event in Earth history.

As concerned with the Glenisla trackway of Australia [22] (G on Fig. 1), it has parallel tracks like some of the Zachelmie tracks of Poland [11]. For some authors (e.g. [40]), the tetrapod interpretation of the Glenisla trackway is very doubtful due to the lack of symmetry of the trackway and the absence of clear alternation in its supposed manus and pes tracks; nevertheless we added it in our Figure 1 because of the possibility that it is of a tetrapod. Even without including the Glenisla trackway in our figure, the radiation of early tetrapods is probably within the Early Devonian after the presently oldest known remains [11] (Z on Fig. 1). Including the Glenisla trackway extends the earliest occurrence of tetrapods near to the base of the Devonian. This has impact on the shape of the elpistostegid-tetrapod cladogram when drawn in front of the geologic time-scale (after [11]), by increasing the ghost range of elpistostegids (a ghost range is an interval of geological time where a fossil lineage *should* exist, but for which there is no direct evidence) down to the Silurian/Devonian boundary at ca. 416 MYA (Fig. 1). The abundance of oxygen during the Early Devonian could have led to the “elpistostegid-tetrapod changeover”. The higher oxygen levels in the marine environments would have helped the tetrapods to evolve into larger organisms. This could mean that the “changeover” occurred during the arthropod terrestrialization unlike previously thought [24]. The higher oxygen levels suggest that earliest tetrapods never needed to breathe oxygen from the air. This feature might have evolved later when the oxygen levels were lower during the Late Devonian and Early Carboniferous (Fig. 1). In the Silurian, vertebrates (fishes) were generally smaller than in the Early Devonian when larger sizes were developed by both agnathans and jawed fishes, including predatory placoderms and sarcopterygians. The increase in biodiversity (number of genera, Fig. 1) and body size of marine invertebrates suggests almost surely that oxygen levels in the atmosphere and the marine environments did increase during the Early Devonian [26], [27], [38]. So, our hypothesis concerning tetrapods, even if it is highly speculative, does fit more global results on terrestrial and marine biodiversity in general, and in the Devonian in particular.

Another global event occurred in the Early Devonian, that is a relative lowering of sea levels that began in the late Silurian through the late Early Devonian [41]. But the higher oxygen levels means that even shallow marine regions were well oxygenated. These regions could be suitable regions for evolution of tetrapods where “walking” on the bottom or in water would prove useful (see recent results on “walking” chondrichthyans, e.g., [42], [43]); these results let thinking that “walking has evolved many times among different lineages of benthic fishes” (in [43]). Walking is energetically less expensive than swimming and walking is used by thorny skates to capture live prey [43]. Such indications provide more possible reasons for evolution of tetrapods in a shallow marine environment. Generally larger organisms have higher metabolic rates [44] and the increase in efficiency of the proteins could be because the evolution of such species with larger mass can only occur during times of higher oxygen levels.

It may happen in the future that, after revision of the paleobiological databases, the Early Devonian biodiversification event has been as important as the Great Ordovician Biodiversification Event (GOBE: [45], [46]) for Paleozoic life. This is supported by the interpretation of Klug et al. [47] who speak of the Devonian Nekton Revolution (DNR) for the re-organization of marine food webs in the Devonian. The nekton revolution of vertebrates did occur earlier in the Silurian [48], but the elpistostegid-tetrapod transition would participate of the early phase of the DNR and the Predation Revolution of vertebrates [48] at a time of high oxygen level. However, we must keep in mind that such scenarios linking global environmental physical factors (such as the atmospheric and oceanic oxygen rate) with development of life on Earth are a pure practice of uniformitarianism in Earth sciences. “Hypotheses linking evolutionary phenomena to atmospheric oxygen levels can be frustratingly difficult to disprove” [49], and “the fundamentally nonuniformitarian nature of Paleozoic and Proterozoic marine ecology must be taken into account” [50].

### Conclusions

Here we suggest that the co-occurrence of a series of bio-events and physical properties of the oceans on Earth during the Early Devonian is not merely a coincidence, but reflects a global re-arrangement of the biosphere. An increase in oxygen is likely to have occurred during the Early Devonian. It would have triggered the emergence of tetrapods in shallow marine environments, where “walking” on the bottom or in the water would have given them advantage in terms of energetic expenses and predation over other fishes. These shallow marine environments could have also proved as ideal regions for the growth of young tetrapods since they could have had fewer predators. We also conclude from the molecular data that lungfishes are much closer to tetrapods than to coelacanths, a result that is not in contradiction with most morphology-based phylogenetic analyses, although it would be hard to pin point and show that these changes shown in the phylogenetic analysis did occur only in the Early Devonian, it is very much a possibility that some of the changes did occur during the Early Devonian. Scenarios such as the ones described above [38], [39], [45], [46], [47] are attractive and represent possible solutions to the relation of global environmental factors and the development of life on Earth. This conclusion is applicable to most, if not all, geobiological scenarios through Earth's history [48].

## Supporting Information

Text S1 References and definitions for the 20 physicochemical [amino acid] properties in TreeSAAP.
(DOC)

Table S1 Species used in the molecular adaptation study.
(XLS)

Table S2 Similar amino acid sites between the different groups of sarcopterygians which have been affected by positive-destabilizing during cladogenesis.
(XLS)
