# Supplementary material for: Rise of the Earliest Tetrapods: An Early Devonian Origin from Marine Environment
Source: PLoS One. 2011 Jul 14;6(7):e22136. doi: 10.1371/journal.pone.0022136 (PMC3136505; doi:10.1371/journal.pone.0022136)
Supplement: Text S1 — References and definitions for the 20 physicochemical [amino acid] properties in TreeSAAP. (DOC) [file pone.0022136.s001.doc]

**References and definitions for the 20 physicochemical [amino acid] properties in TreeSAAP**

**Alpha-helical Tendency**

AAindex ID: CHOP780201

AAindex name: “Normalized frequency of alpha-helix (Chou-Fasman, 1978b)”

G&P93 abbreviation: *P_α_*

**Chou & Fasman, 1978** (AAindex ref)**:**

Chou, PY and GD Fasman (1978) Prediction of the secondary structure of proteins from their amino acid sequence. *Advances in Enzymology and Related Areas of Molecular Biology* **47:**45-148. (PMID: 364941)

**Average Number of Surrounding Residues**

AAindex ID: PONP800108

AAindex name: “Average number of surrounding residues (Ponnuswamy et al., 1980)”

G&P93 abbreviation: *N_s_*

**Ponnuswamy et al., 1980** (AAindex ref)**:**

Ponnuswamy, PK, M Prabhakaran, and P Manavalan (1980) Hydrophobic packing and spatial arrangement of amino acid residues in globular proteins. *Biochimica et Biophysica Acta* **623:**301-316. (PMID: 7397216)

“In general, the number of residues surrounding a residue within the effective distance of influence is the important factor for the change in the surrounding hydrophobicity indices of the residues.”

**Buriedness**

AAindex ID:

AAindex name:

G&P93 abbreviation: *B_r_*

**Ponnuswamy et al., 1980:**

Ponnuswamy, PH, M Prabhakaran, and P Manavalan (1980) Hydrophobic packing and spatial arrangement of amino acid residues in globular proteins. *Biochimica et Biophysica Acta* **623:**301-316.

Buried nature of residues discussed in Ponnuswamy et al., 1980, but values are different than those used by Gromiha & Ponnuswamy, 1993. Consider changing to this apparently more accepted version of buriedness.

**Gromiha & Ponnuswamy, 1993:**

Gromiha, MM and PK Ponnuswamy (1993) Relationship between amino acid properties and protein compressibility. *Journal of Theoretical Biology* **165:**87-100.

Values given in Gromiha & Ponnuswamy, 1993, but property is not defined here.

**Chromatographic Index**

AAindex ID: ZIMJ680105

AAindex name: “RF rank (Zimmerman et al., 1968)”

G&P93 abbreviation: *R_F_*

**Zimmerman et al., 1968** (AAindex ref)**:**

Zimmerman, JM, N Eliezer, and R Simha (1968) The characterization of amino acid sequences in proteins by statistical methods. *Journal of Theoretical Biology* **21:**170-201. (PMID: 5700434)

“In searching for a classification which might provide a composite evaluation of amino acid interactions one is led to the partitioning of these residues in solvent systems by means of paper chromatography. For example, Woese (1965) has recently shown that there exists a remarkable correlation between the *R_F_* values of the amino acids and the nucleotide codons associated with each of these residues. Although the amino acids have been separated by paper chromatography in a variety of solvent systems, none of these can be considered to resemble the biological environment…Three candidate *R_F_* indices were developed for possible use in the sequence analyses as follows:”

… …

“(3) For each solvent system the amino acids were ranked starting with one for the lowest value and proceeding upward. The ranked values were then averaged for each residue over all seven solvent systems.”

Woese, CR (1965) Order in the genetic code. *Proceedings of the National Academy of Sciences, USA* **54:**71-75.

**Prabhakaran & Ponnuswamy, 1979:**

Prabhakaran, M and PK Ponnuswamy (1979) The spatial distribution of physical, chemical, energetic and conformational properties of amino acid residues in globular proteins. *Journal of Theoretical Biology* **80:**485-504.

“The chromatographic index of an amino acid specifies its characteristic migration rate in a solvent-absorbant system which is a measure of the composite nature of interaction of solute, solvent and absorbant. Different kinds of *R_F_* indices are defined in literature, of which we have taken the average ranked value for each molecule over seven solvent systems. It was shown by Woese (1965) that there exists a remarkable correlation between the *R_F_* values of the amino acids and the nucleotide codons associated with each of these residues.”

**Coil Tendency**

AAindex ID: CHAM830101

AAindex name: “The Chou-Fasman parameter of the coil conformation (Charton-Charton, 1983)”

G&P93 abbreviation: *P_c_*

**Charton & Charton, 1983** (AAindex ref)**:**

Charton, M and B Charton (1983) The dependence of the Chou-Fasman parameters on amino acid side chain structure. *Journal of Theoretical Biology* **102:**121-134. [ref. on AAindex – *JTB* **111:**447-450 – is incorrect] (PMID: 6876837)

“Turn and coil parameters show little or no difference in their dependence which is different from that of α-helix and in some ways almost reciprocal. Factors which increase the probability of finding an amino acid residue in an α-helix usually decrease the probability of finding it in a coil or turn.”

… …

“The structural dependence of the probability of a residue being in a coil parallels the probability of it being in a turn, except that the presence or absence of an ionic charge has no influence.”

**Compressibility**

AAindex ID:

AAindex name:

G&P93 abbreviation: *K*^0^

**Gromiha & Ponnuswamy, 1993:**

Gromiha, MM and PK Ponnuswamy (1993) Relationship between amino acid properties and protein compressibility. *Journal of Theoretical Biology* **165:**87-100.

“Specific compressibility (*K*^0^) is the reciprocal of bulk modulus, i.e. the relative increase in the volume of the system per unit decrease in pressure…” Units: m^3^ mol^-1^ Pa^-1^ (x10^-15^).

**Equilibrium Constant**

AAindex ID: JOND750102

AAindex name: “pK (-COOH) (Jones, 1975)” – long name: “Equilibrium constant with reference to the ionization property of the -COOH group”

G&P93 abbreviation: *pK’*; also referred to as *pK*_1_

**Jones, 1975** (AAindex ref)**:**

Jones, DD (1975) Amino acid properties and side-chain orientation in proteins: A cross correlation approach. *Journal of Theoretical Biology* **50:**167-183. (PMID: 1127956)

McMeekin, TL, ML Groves, and NJ Hipp (1964) Pp. 54 in *Amino Acids and Serum Proteins* (Stekol, JA, ed.). American Chemical Society, Washington, DC.

**Prabhakaran & Ponnuswamy, 1979:**

Prabhakaran, M and PK Ponnuswamy (1979) The spatial distribution of physical, chemical, energetic and conformational properties of amino acid residues in globular proteins. *Journal of Theoretical Biology* **80:**485-504.

“pK’…the pK value of the COOH group…represents [the ionizable character] of the carboxyl group…”

**Helical Contact Area**

AAindex ID:

AAindex name:

G&P93 abbreviation: *C_a_*

**Richmond & Richards, 1978:**

Richmond, TJ and FM Richards (1978) Packing of α-helices: Geometrical constraints and contact areas. *Journal of Molecular Biology* **119:**537-555.

“For the smaller non-polar residues this number is equal to the maximum area loss that could occur in going from an isolated α-helix to a fully buried environment in the complex. For the larger non-polar residues only a fraction of this value is used since they can never be totally buried in a single interaction site. The polar residues are difficult to evaluate. Along with non-polar atoms capable of favorable energy contributions, polar groups in a given residue may also be buried with a presumed adverse effect on the interaction energy. Because of these competing energetic effects, for many of these residues a zero energy contribution has been assumed and a zero area change assigned.”

**Muthusamy & Ponnuswamy, 1990:**

Muthusamy, R and PK Ponnuswamy (1990) Variation of amino acid properties in protein secondary structures, α-helices and β-strands. *International Journal of Peptide and Protein Research* **35:**378-395.

“The standard α-helical and β-strand contact areas denoted, respectively, as C_αh_ and C_βh_, were taken from Richmond & Richards [11].”

[11] Richmond, TJ and FM Richards (1978) Packing of α-helices: Geometrical constraints and contact areas. *Journal of Molecular Biology* **119:**537-555.

**Gromiha & Ponnuswamy, 1993:**

Gromiha, MM and PK Ponnuswamy (1993) Relationship between amino acid properties and protein compressibility. *Journal of Theoretical Biology* **165:**87-100.

**Hydropathy**

AAindex ID: KYTJ820101

AAindex name: “Hydropathy index (Kyte-Doolittle, 1982)”

**Kyte & Doolittle, 1982** (AAindex ref)**:**

Kyte, J and RF Doolittle (1982) A simple method for displaying the hydropathic character of a protein. *Journal of Molecular Biology* **157:**105-132. (PMID: 7108955)

“Ideally, the most satisfying way to determine the hydrophobic or hydrophilic inclinations of a given amino acid side-chain (i.e. its hydropathy^†^) would be to measure its partition coefficient between water and a non-interacting, isotropic phase and to calculate from that partition coefficient a transfer free energy.”

… …

“^†^ Since hydrophilicity and hydrophobicity are no more than the two extremes of a spectrum, a term that defines that spectrum would be as useful as either, just as the term light is as useful as violet light or red light. Hydropathy (strong feeling about water) has been chosen for this purpose.”

… …

“We have used both the water-vapor transfer free energies and the interior-exterior distribution of amino acid side-chains determined by Chothia (1976) in assigning the final hydropathy values (Table 2). Results presented later in this paper indicate clearly that the number in the second place of the hydropathy values is of little consequence to the hydropathy profiles, and as a result we did not hesitate to adjust the values subjectively when only this level of accuracy was in question. Nevertheless, we tried to derive the best numbers we could from the data listed in the last 3 columns of Table 2 [*∆G°*_transfer_ (kcal mol^-1^) of (1) water into condensed vapor, (2) water into ethanol, and (3) ethanol into condensed vapor]. The hydropathy values for **valine**, **phenylalanine**, **threonine**, **serine** and **histidine** were simple averages of the 3 other numbers in the Table. When 1 of the 3 numbers for a given amino acid was significantly different from the other 2, the mean of the other 2 was used. This was done for **cysteine/cystine**, **methionine** and **isoleucine**. After a good deal of futile discussion concerning the differences among **glutamic acid**, **aspartic acid**, **asparagines** and **glutamine**, we came to the conclusion that they all had indistinguishable hydropathies and set their hydropathy value by averaging all of the normalized water-vapor transfer free energies and the normalized fractions of side chains 100% buried. Because the structural information was so uncertain, **tryptophan** was simply assigned its normalized transfer free energy. **Glycine** was arbitrarily assigned the hydropathy value which was the weighted mean of the hydropathy values for all of the sequences in our data base because it was clear from a careful analysis of the actual distribution of **glycine** that it is no hydropathic; that is to say, it does not have strong feelings about water. One the basis of both the transfer free energy scale and the fraction buried, **alanine** ought to be more hydrophobic on our scale, its value exceeding that of **leucine**. We find it difficult to accept that a single methyl group can elicit more hydrophobic force than a cluster of 4 methyl groups, and for that reason we have arbitrarily lowered the hydropathy value of the **alanine** side-chain to a point half-way between the hydropathy value of **glycine** and the value determined for **alanine** when the transfer energy and its distribution is used. No suitable model exists for **proline**, and in terms of its tendency to become buried it is fairly hydrophilic. Its hydropathy value was made somewhat more hydrophobic than this consideration because of its 3 methylene groups. The hydropathy value for **arginine** was arbitrarily assigned to the lowest point of the scale. Because it was difficult to accept the fact that **tyrosine** is a hydrophilic amino acid, even though the available data in Table 2 indicate that it is, its hydropathy value was subjectively raised to one closer to the water-vapor transfer free energy than the structural data would have yielded. Similarly, the hydropathy value for **leucine** was also raised above the average of the structural data and the transfer free energy, and the hydropathy value for **lysine** was lowered. None of the last 3 adjustments, the result of personal bias and heated discussion between the authors, affects the hydropathy profiles in any significant way.”

Chothia, C (1976) The nature of the accessible and buried surfaces in proteins. *Journal of Molecular Biology* **105:**1-14.

**Isoelectric Point**

AAindex ID: ZIMJ680104

AAindex name: “Isoelectric point (Zimmerman et al., 1968)”

**Zimmerman et al., 1968** (AAindex ref)**:**

Zimmerman, JM, N Eliezer, and R Simha (1968) The characterization of amino acid sequences in proteins by statistical methods. *Journal of Theoretical Biology* **21:**170-201. (PMID: 5700434)

**Prabhakaran & Ponnuswamy, 1979:**

Prabhakaran, M and PK Ponnuswamy (1979) The spatial distribution of physical, chemical, energetic and conformational properties of amino acid residues in globular proteins. *Journal of Theoretical Biology* **80:**485-504.

“pH_i_…the isoionic point of the free amino acid…includes the ionizable character of either the sidechain or amino group plus the carboxyl group of the molecule…”

**Mean r.m.s. Fluctuational Displacement**

AAindex ID:

AAindex name: - r.m.s. most likely is an acronym for “root mean square”

G&P93 abbreviation: *F*

**Bhaskaran & Ponnuswamy, 1984:**

Bhaskaran, R and PK Ponnuswamy (1984) Dynamics of amino acid residues in globular proteins. *International Journal of Peptide and Protein Research* **24:**180-191.

“X-ray diffraction data on the atomic positions coupled with the assumption that the maximum permitted residue-displacement equals one-tenth of the magnitude of the maximum radius vector of the protein form the basis for this method of study.”

… …

“This parameter relates the amount of displacement of any element with its distance from the centroid of the protein. Each protein is characterized by its specific internal residue-distribution and associated fluctuational situation. To reflect this, we incorporate boundary conditions and solve eqn. 5 [from Ponnuswamy and Bhaskaran (1984)] to obtain displacements for all the residues of the protein within a specific range-between u_i_ in the neighborhood to zero (for the residue at the centroid) and u_max_, the 10% of the maximum value of the radius vector for the protein.”

Ponnuswamy, PK and R Bhaskaran (1984) *International Journal of Peptide and Protein Research* **24:**168-179. [i.e., the article immediately preceding the one in which this article is cited in the same issue – title unknown at this point.]

**Muthusamy & Ponnuswamy, 1990:**

Muthusamy, R and PK Ponnuswamy (1990) Variation of amino acid properties in protein secondary structures, α-helices and β-strands. *International Journal of Peptide and Protein Research* **35:**378-395.

“The mean r.m.s. displacements of the amino acid residues computed by Bhaskaran & Ponnuswamy [12] in their study on the dynamics of globular proteins were taken to be the values of the fluctuational parameter F_1_.”

[12] Bhaskaran, R and PK Ponnuswamy (1984) Dynamics of amino acid residues in globular proteins. *International Journal of Peptide and Protein Research* **24:**180-191.

**Gromiha & Ponnuswamy, 1993:**

Gromiha, MM and PK Ponnuswamy (1993) Relationship between amino acid properties and protein compressibility. *Journal of Theoretical Biology* **165:**87-100.

**Normalized Consensus Hydrophobicity**

AAindex ID:

AAindex name:

G&P93 abbreviation: *H_nc_*

**Muthusamy & Ponnuswamy, 1990:**

Muthusamy, R and PK Ponnuswamy (1990) Variation of amino acid properties in protein secondary structures, α-helices and β-strands. *International Journal of Peptide and Protein Research* **35:**378-395.

Although this paper lists values for *H_nc_*, it claims that they originated with Prabhakaran and Ponnuswamy (1979). Upon close exmination of the Prabhakaran and Ponnuswamy paper, however, there is no mention of any normalized consensus measure of hydrophobicity. That being said, the 1979 paper does present two other measures of hydrophobicity: (1) thermodynamic transfer hydrophobicity, and (2) surrounding hydrophobicity. Perhaps normalized consensus hydrophobicity is calculated from these other two measures. If this is the case, there is no indication of how this was accomplished.

Prabhakaran, M and PK Ponnuswamy (1979) The spatial distribution of physical, chemical, energetic and conformational properties of amino acid residues in globular proteins. *Journal of Theoretical Biology* **80:**485-504.

**Gromiha & Ponnuswamy, 1993:**

Gromiha, MM and PK Ponnuswamy (1993) Relationship between amino acid properties and protein compressibility. *Journal of Theoretical Biology* **165:**87-100.

**Polarity**

AAindex ID: GRAR740102

AAindex name: “Polarity (Grantham, 1974)”

Grantham74 abbreviation: *p*

**Grantham, 1974** (AAindex ref)**:**

Grantham, R (1974) Amino acid difference formula to help explain protein evolution. *Science* **185:**862-864. (PMID: 4843792)

“Polarity is averaged: *p* = (*PR* + *PA*)/2, where *PR* is the ‘polar requirement’…and *PA* (adjusted to same scale as *PR*) = 13.66 – 14.85 *R_f_*, where *R_f_* is the amino acid mobility…”

**Power to be at the N-terminus of the Alpha-helix**

AAindex ID:

AAindex name:

C&F74 abbreviation: *f*_hN_/*f*

**Chou & Fasman, 1974:**

Chou, PY and GD Fasman (1974) Conformational parameters for amino acids in helical, β-sheet, and random coil regions calculated from proteins. *Biochemistry* **13:**211-222.

[See *f*_hN_ in Table IV of this publication; the actual value of this property is normalized *f*_hN_/*f* – the three “power to be” properties used in TreeSAAP were subsequently mislabeled in later publications, thus α_c_ in Gromiha and Ponnuswamy (1993) is really power to be at the N-terminus of the alpha-helix. Referred to in Chou and Fasman (1974) as the normalized frequency of residues in the N-terminal helix region.]

**Prabhakaran & Ponnuswamy, 1979:**

Prabhakaran, M and PK Ponnuswamy (1979) The spatial distribution of physical, chemical, energetic and conformational properties of amino acid residues in globular proteins. *Journal of Theoretical Biology* **80:**485-504.

“The helix adopting power of a residue is again divided into three types, namely, power to be at (i) the *N*-terminus, (ii) the *C*-terminus and (iii) the middle position of an α-helical section. The parameters representing these conformational properties of the residues were determined by Chou & Fasman (1974) from an analysis of a number of crystalline globular proteins.”

**Short and Medium Range Non-bonded Energy**

AAindex ID: OOBM770102

AAindex name: “Short and medium range non-bonded energy per atom (Oobatake-Ooi, 1977)”

G&P93 abbreviation: *E_sm_*

**Oobatake & Ooi, 1977** (AAindex ref)**:**

Oobatake, M and T Ooi (1977) An analysis of non-bonded energy of proteins. *Journal of Theoretical Biology* **67:**567-584. (PMID: 904331)

“The short-range interaction is defined as the energy between main chain atoms of a residue and its own side chain atoms, the medium-range interaction as that between two residues located within 10 residues along the chain, and the long-range interaction as that between two residues separated further.”

**Prabhakaran & Ponnuswamy, 1979:**

Prabhakaran, M and PK Ponnuswamy (1979) The spatial distribution of physical, chemical, energetic and conformational properties of amino acid residues in globular proteins. *Journal of Theoretical Biology* **80:**485-504.

“The energy of two interacting residues is conveniently classified into three categories depending upon the number of residues existing between the two residues along the sequence: short-range, medium-range and long-range (Oobatake & Ooi, 1977). The short-range interaction is defined as the energy between the main chain atoms of the residue and its own sidechain atoms, the medium-range interaction as that between the two residues located within ten residues separated further. Oobatake and Ooi calculated the non-bonded energy per atom of the residue in terms of these three classifications for 16 proteins using the atomic co-ordinates obtained by X-ray crystallography and determined the average contribution in each category for each of the amino acid residues. They have shown that the sum of short-range and medium-range interactions amounts to 72.5% of the total energy, suggesting that the interactions in these ranges are important for the folding of the polypeptide chain. They used the average energy of amino acid residues to estimate the local energy of a segment in protein molecules.”

**Solvent Accessible Reduction Ratio**

AAindex ID: PONP800107

AAindex name: “Accessibility reduction ratio (Ponnuswamy et al., 1980)”

G&P93 abbreviation: *R_a_*

**Ponnuswamy et al., 1980** (AAindex ref)**:**

Ponnuswamy, PK, M Prabhakaran, and P Manavalan (1980) Hydrophobic packing and spatial arrangement of amino acid residues in globular proteins. *Biochimica et Biophysica Acta* **623:**301-316. (PMID: 7397216)

“An interesting aspect of the present study on surrounding hydrophobicity is that the results could be compared with the results of solvent accessibility studies made on protein molecules, by Lee and Richards [20] and by Chothia [9]. These authors calculated the decrease in solvent accessibility for an amino acid residue when the protein molecule moves from a hypothetically extended state to the native folded state. We have computed here the gain in the surrounding hydrophobicity for an amino acid residue when the protein molecule is transferred from a similar extended state to the native folded state, and hence the reduction in the solvent accessibility and the gain in the surrounding hydrophobicity have an inverse relationship. In Table I the solvent accessibility reduction ratios $\left\langle R_{a}\right\rangle$, and the surrounding hydrophobicity gain ratios $\left\langle G_{h}\right\rangle$, for the 20 amino acid residues are given for comparison.”

[9] Chothia, C (1976) The nature of the accessible and buried surfaces in proteins. *Journal of Molecular Biology* **105:**1-14.

[20] Lee, B and FM Richards (1971) The interpretation of protein structures: Estimation of static accessibility. *Journal of Molecular Biology* **55:**379-400.

**Surrounding Hydrophobicity**

AAindex ID:

AAindex name:

G&P93 abbreviation: *H_p_*

**Prabhakaran & Ponnuswamy 1979:**

Prabhakaran, M and PK Ponnuswamy (1979) The spatial distribution of physical, chemical, energetic and conformational properties of amino acid residues in globular proteins. *Journal of Theoretical Biology* **80:**485-504.

“The surrounding hydrophobic index or the bulk hydrophobicity of an amino acid residue…is a parameter determined by Manavalan & Ponnuswamy (1978) taking into consideration that the medium of protein environment in which the residue is embedded is very different from that used by Tanford [1962]. This parameter, determined by constructing an optimum sphere of 8 Å radius around a residue in protein crystals and by summing up the Tanford-Jones [1975] hydrophobic indices assigned to all the residues within this sphere was demonstrated by the authors to correlate with the extent of buriedness of the residue in protein crystals in a far better way than the corresponding Tanford-Jones hydrophobic index [thermodynamic transfer hydrophobicity] of the residue.”

**Ponnuswamy et al., 1980:**

Ponnuswamy, PK, M Prabhakaran, and P Manavalan (1980) Hydrophobic packing and spatial arrangement of amino acid residues in globular proteins. *Biochimica et Biophysica Acta* **623:**301-316. (different values)

“We define ‘the surrounding hydrophobicity’ of a residue as the sum of the hydrophobic indices assigned to the various residues that appear within an 8 Å radius volume in the protein crystal [15].”

“…the parameter ‘surrounding hydrophobicity’ could be fruitfully used to describe the internal packing arrangements of residues in globular proteins.”

[15] Manavalan, P and PK Ponnuswamy (1978) Hyrophobic character of amino acid residues in globular proteins. *Nature* **275:**673-674. (different values)

**Gromiha & Ponnuswamy, 1993:**

Gromiha, MM and PK Ponnuswamy (1993) Relationship between amino acid properties and protein compressibility. *Journal of Theoretical Biology* **165:**87-100. (not defined here)

**Magyar et al., 2005:**

Magyar, C, MM Gromiha, G Pujadas, GE Tusnády, and I Simon (2005) SRide: A server for identifying stabilizing residues in proteins. *Nucleic Acids Research* **33:**W303-W305. (values not listed)

“Surrounding hydrophobicity of a residue *I* [*H*_P_(*i*)] is calculated as the sum of hydrophobic indices, obtained from thermodynamic transfer experiments, of residues whose C_α_ atoms are within the distance of 8 Å from the C_α_ atom of residue *i*:

$$H_{\text{P}}\left(i\right)=\sum_{j=1}^{20} n_{ij}h_{j}$$

where *n_ij_* is the total number of surrounding residues of type *j* around residue *i* of the protein, and *h_j_* is the hydrophobic index of residue type *j*, in kcal/mol listed in [18].”

[18] Nozaki, Y and C Tanford (1971) The solubility of amino acids and two glycine peptides in aqueous ethanol and dioxane solutions. Establishment of a hydrophobicity scale. *Journal of Biological Chemistry* **246:**2211-2217.

**Thermodynamic Transfer Hydrophobicity**

AAindex ID: JOND750101

AAindex name: “Hydrophobicity (Jones, 1975)”

G&P93 abbreviation: *H_t_*

**Zimmerman et al., 1968:**

Zimmerman, JM, N Eliezer, and R Simha (1968) The characterization of amino acid sequences in proteins by statistical methods. *Journal of Theoretical Biology* **21:**170-201.

“The last classification employed was a hydrophobicity index based on solubility data of Tanford (1962). As pointed out earlier this characteristic may play a very significant role in the alignment of amino acids in the three-dimensional configuration.”

Tanford, C (1962) Contribution of hydrophobic interactions to the stability of the globular conformation of proteins. *Journal of the American Chemical Society* **84:**4240.

**Nozaki & Tanford, 1971:**

Nozaki, Y and C Tanford (1971) The solubility of amino acids and two glycine peptides in aqueous ethanol and dioxane solutions. Establishment of a hydrophobicity scale. *Journal of Biological Chemistry* **246:**2211-2217.

“For those amino acid side chains that appear to be predominantly hydrophobic (i.e. have similar contact free energies in a variety of media other than water, and a large positive contact free energy in water, relative to all such other media) the difference between solvation free energies in water and in 100% ethanol or other nonaqueous media may be considered as establishing a *hydrophobicity scale*, which can be regarded, for example, as a measure of the tendency of that side chain to be located in the interior of a native protein molecule [14].”

[14] Bigelow, CC (1967) On the average hydrophobicity of proteins and the relation between it and protein structure. *Journal of Theoretical Biology* **16:**187.

**Jones, 1975** (AAindex ref)**:**

Jones, DD (1975) Amino acid properties and side-chain orientation in proteins: A cross correlation approach. *Journal of Theoretical Biology* **50:**167-183. (PMID:1127956)

“The hydrophobicity scale was revised in the light of more recent experimental data of Nozaki & Tanford (1971). Except for leucine, serine, tryptophan and phenylalanine, the values based on the new data do not differ greatly from the values used by Zimmerman et al. (1968). For amino acids that were not studies by Nozaki & Tanford, namely, the charged amino acids, the values used by Zimmerman et al. (1968) were adjusted to the new scale by a linear least-squares regression.”

**Prabhakaran & Ponnuswamy, 1979:**

Prabhakaran, M and PK Ponnuswamy (1979) The spatial distribution of physical, chemical, energetic and conformational properties of amino acid residues in globular proteins. *Journal of Theoretical Biology* **80:**485-504.

“The parameter hydrophobic index, first quantitatively assessed for each of the nonpolar amino acids from thermodynamic measurements by Tanford (1962) and extrapolated later to rest of the amino acids by Jones (1975) represents the intrinsic hydrophobic character of the sidechain of the free amino acid with reference to the simple organic surrounding medium used in the original experiments by Tanford.”

**Total Non-bonded Energy**

AAindex ID: OOBM770101

AAindex name: “Average non-bonded energy per atom (Oobatake-Ooi, 1977)”

G&P93 abbreviation: *E_t_*

**Oobatake & Ooi, 1977** (AAindex ref)**:**

Oobatake, M and T Ooi (1977) An analysis of non-bonded energy of proteins. *Journal of Theoretical Biology* **67:**567-584. (PMID:904331)

“The short-range interaction is defined as the energy between main chain atoms of a residue and its own side chain atoms, the medium-range interaction as that between two residues located within 10 residues along the chain, and the long-range interaction as that between two residues separated further.” (Total non-bonded energy is the sum of short, medium, and long range non-bonded energy.)

**Turn Tendency**

AAindex ID: CHOP780101

AAindex name: “Normalized frequency of beta-turn (Chou-Fasman, 1978a)”

G&P93 abbreviation: *P_t_*

**Chou & Fasman, 1978** (AAindex ref)**:**

Chou, PY and GD Fasman (1978) Empirical predictions of protein conformation. *Annual Review of Biochemistry* **47:**251-276. (PMID: 354496)
